# Sporadic Case of Acrokeratosis Verruciformis of Hopf With Unusual Clinical Presentation: A Case Report and Review of the Literature

**DOI:** 10.7759/cureus.91671

**Published:** 2025-09-05

**Authors:** Abraham Isaí Cabello-Hernández, Ximena Rodríguez-Rangel, Alondra Saray Polanco-Llanes, Sonia Toussaint-Caire, Judith Domínguez-Cherit

**Affiliations:** 1 Department of Dermatology, Hospital General "Dr. Manuel Gea González", Mexico City, MEX; 2 Department of Dermatopathology, Hospital General "Dr. Manuel Gea González", Mexico City, MEX; 3 Department of Dermatologic Surgery, Hospital General "Dr. Manuel Gea González", Mexico City, MEX

**Keywords:** acrokeratosis verruciformis of hopf, autosomal dominant inheritance, darier’s disease, epidermodysplasia verruciformis, genodermatosis

## Abstract

Objective: This study aims to report a sporadic case of acrokeratosis verruciformis of Hopf (or acrokeratosis verruciformis (AKV)) with an unusual clinical presentation, highlighting its diagnostic challenges and key distinguishing features.

Case report: A 54-year-old female patient with no relevant medical history presented with multiple skin-colored, flat-topped, hyperkeratotic papules on the dorsal aspects of the hands since childhood, and onychopathy for 12 years. Nail changes included thickening of the nail plate and subungual hemorrhages. She presented a soft, warty pink-colored plaque in the right inguinal region. Dermoscopy of the dorsum of the hand showed a white network and papules with a central homogeneous area and a peripheral cobblestone appearance. A biopsy was performed and showed hyperorthokeratosis and epidermal hyperplasia with focal papillomatosis. Treatment was started with keratolytics. The patient has been under follow-up by our department for three months with partial improvement of the lesions.

Discussion: AKV, a rare genodermatosis, presents a diagnostic challenge due to its subtle onset, relying primarily on clinical presentation and histopathological characteristics. Genetic studies can aid in identifying underlying mutations, with Darier's disease being the main differential diagnosis. Management typically involves keratolytic agents and retinoids, though superficial ablation is considered first-line treatment.

Conclusion: AKV should be suspected in patients presenting with numerous flat, skin-colored, polygonal papules on acral areas and long-standing onychopathy. This case emphasizes the critical role of comprehensive clinical assessment and histopathological study for accurate diagnosis, especially given its unusual manifestations.

## Introduction

Acrokeratosis verruciformis of Hopf (or acrokeratosis verruciformis (AKV)) is a rare autosomal dominant genodermatosis with a worldwide distribution and without racial predilection [1]. It is characterized by the presence of keratotic papules that are either skin-colored or brown, primarily affecting the dorsum of the hands and feet, although other areas can also be involved [1,2]. Nail changes such as thickening of the nail plate and leukonychia may be observed in some patients. Differential diagnoses to be considered are Darier's disease and epidermodysplasia verruciformis [1]. We describe a case of a patient presenting with AKV with significant nail alterations and unusual involvement of the genitalia and inguinal area. We also emphasize the significance of clinical and histopathological examination in the differential diagnosis. 

## Case presentation

**Figure 1:**
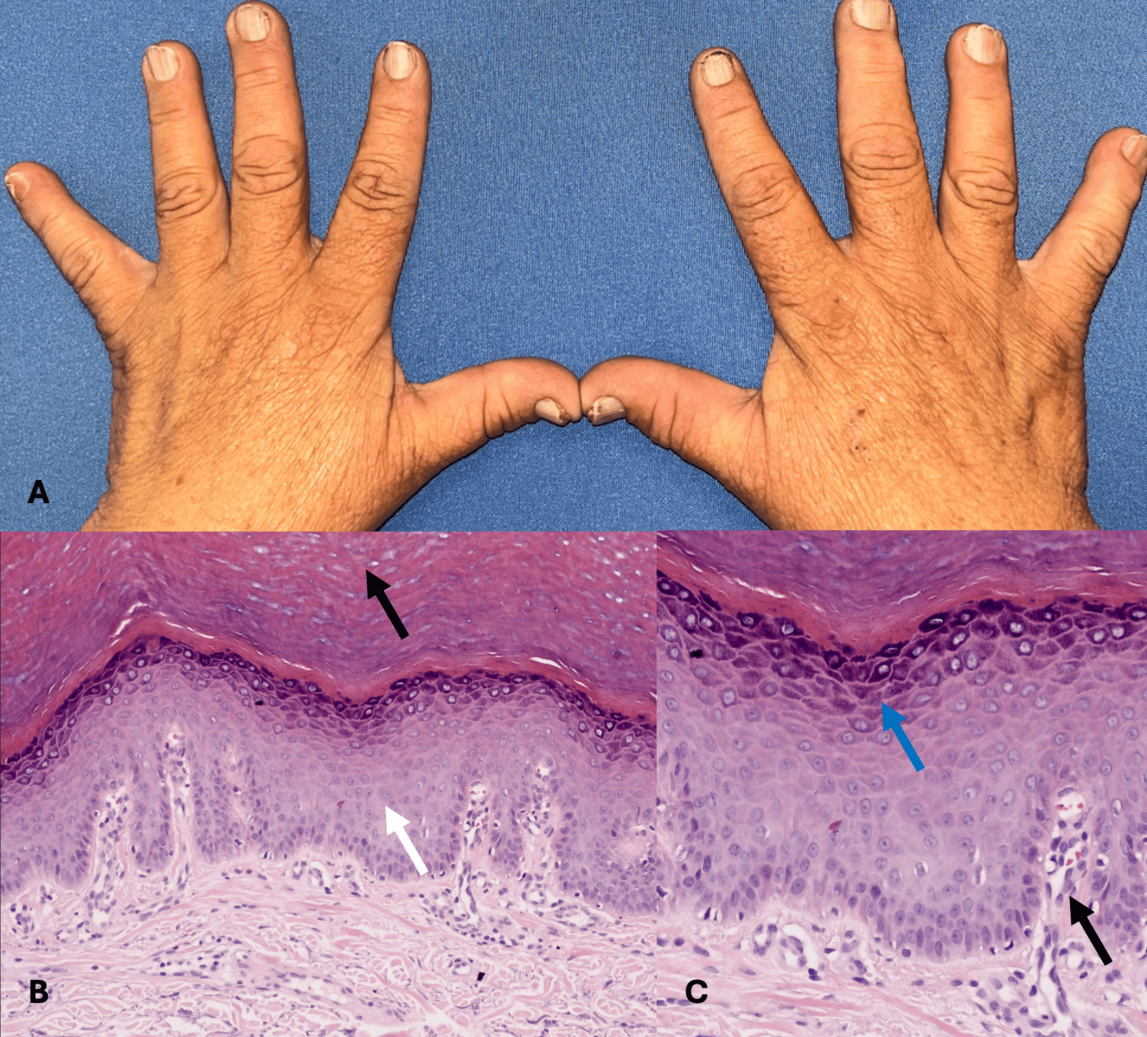
Clinical image and histological image of the dorsum of the hands (A) Dermatosis consisting of multiple flat-topped skin-colored papules of 2 mm in diameter, which coalesce to give a cobblestone appearance. (B) Hyperorthokeratosis (black arrow)and acanthosis (white arrow). (C) Hypergranulosis (blue arrow) and focal papillomatosis (black arrow) (hematoxylin-eosin stain; original magnification B x20, and C, x40)

**Figure 2:**
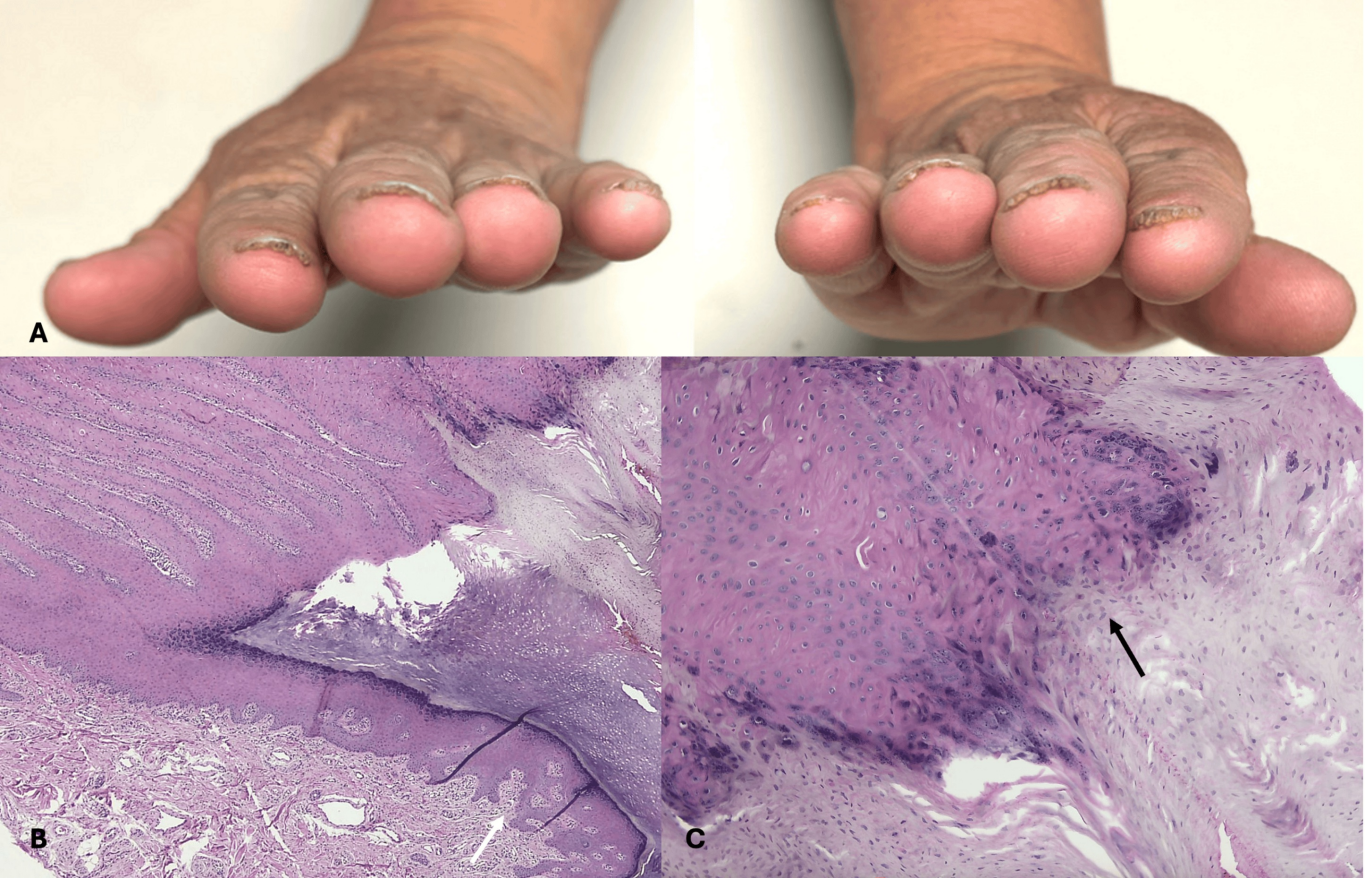
Clinical image and histological image of fingernails (A) Onychopathy of the 10 fingernails, characterized by subungual keratosis and distal onycholysis. (B, C) Epithelial hyperplasia with papillomatosis (white arrow) and hyperparakeratosis of the nail matrix (black arrow) (hematoxylin-eosin stain; original magnification B x20 and C x40)

**Figure 3:**
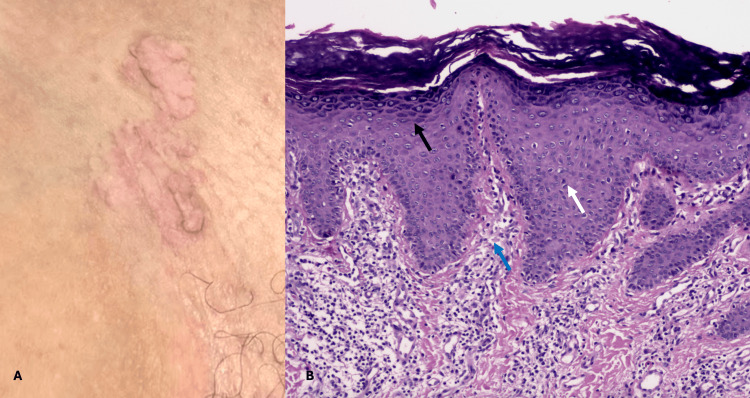
Clinical image and histological image of right inguinal region (A) Soft, warty pink-colored plaque. (B) Epidermal hyperplasia (white arrow) with hypergranulosis (black arrow) and a mild perivascular infiltrate of lymphocytes (blue arrow) (hematoxylin-eosin stain; original magnification x20)

A 54-year-old housewife presented with a disseminated dermatosis on the trunk and the four extremities characterized by a dermatosis consisting of multiple flat-topped skin-colored papules of 2 mm in diameter, which coalesce to give a cobblestone appearance, in the arms, dorsum of the hand, and genitalia (Figure 1A). Lesions on the arms started during childhood, and at 42 years of age, she began with onychopathy of the 10 fingernails, characterized by subungual keratosis and distal onycholysis (Figure 2A). Subsequently, at the age of 52 years, she presented in the right inguinal region, a soft, warty pink-colored plaque (Figure 3A). At the time of evaluation, the patient did not point out any family members affected. Differential diagnoses included nail lichen planus and subungual warts. Dermoscopy of the dorsum of the hand showed a white network and papules with a central homogeneous area and peripheral cobblestone appearance. Onychoscopy revealed subungual hemorrhages on the fingernails bilaterally. Given the thickening of the nail plate, direct examination was performed, which ruled out onychomycosis. 

Biopsies were performed from three sites. Histopathological study of the warty lesion of the right inguinal region showed epidermal hyperplasia with hypergranulosis and a mild perivascular infiltrate of lymphocytes (Figure 2B). Biopsies taken from the papular lesion on the dorsum of the left hand showed hyperorthokeratosis and epidermal hyperplasia with focal papillomatosis compatible with the verrucous lesions associated with AKV (Figure 1B). Likewise, the histopathological study of the fifth fingernail showed epidermal hyperplasia with papillomatosis and hyperparakeratosis of the hyponychium (Figure 3B); a polymerase chain reaction (PCR) test was performed to search for infection with human papillomavirus, which was negative. The genetic study was not feasible; however, based on the clinical and histopathological findings, the diagnosis of AKV was established. The patient was treated with keratolytics and has been under follow-up for three months with partial improvement of symptoms.

## Discussion

AKV is a rare genodermatosis without race predilection, benign, that typically occurs in childhood but may appear as late as the fifth decade of life [1]. This disease has a heterozygous mutation pro602Leu in the ATP2A2 gene on chromosome 12q24, the same gene mutation responsible for Darier's disease [2].​ This mutation has an autosomal dominant pattern of inheritance but incomplete penetrance, which means that a patient will not always have a family history of the disease [1]​. At the time of evaluation, the patient did not point out any family members affected; this can be attributed to its incomplete penetrance. The ATP2A2 gene encodes the sarcoplasmic reticulum Ca2+ ATPase2 pump, which has a role in signalling pathways regulating cell-to-cell adhesion and differentiation of the epidermis. The P602L mutation leads to an inability to transport calcium in the sarcoendoplasmic reticulum calcium ATPase, leading to dyskeratosis [3]. ​ 

This disease is clinically manifested by the presence of multiple flat-topped, polygonal papules, and verrucous plaques. These lesions are firm and skin-colored with friction on the adjacent skin, potentially inducing vesicle formation [1]. ​The predominant distribution is on the dorsal surfaces of the hands and feet; the palmar skin may be thickened and show punctate keratosis, pits, or punctiform breaks in dermatoglyphics, though involvement of the legs, knees, arms, and elbows may occur less frequently. Forehead, scalp, flexures, and oral mucosa are not commonly affected [4]. ​Additional findings can include thickening of the nail plate, leukonychia, and the presence of longitudinal ridges breaking at the distal edge [1]. ​ 

There are two types of AKV, classic and sporadic. Classic AKV usually appears during childhood, and the typical morphology of the lesions is observed on the back of the hands and feet. In contrast, the age of onset of sporadic AKV is much later and may affect other sites such as the face, scalp, and trunk. A positive family history with palmar pits and nail changes is observed in classic AKV, but not in sporadic AKV [5]. ​​ 

Histopathologically, the lesions exhibit "church spire" epidermal papillomatosis, acanthosis, hyperkeratosis, and hypergranulosis without parakeratosis. The rete ridges are slightly elongated and extended to a uniform level. The changes are mainly observed at the epidermal level, but a dermal infiltrate may be observed [6]. 

Dermatoscopic findings may also guide the diagnosis. The white network corresponds to the combined hyperkeratosis and acanthosis. The holes correlate with the lower foci of the epithelial papillomatosis and the cobblestone appearance and a central white homogenous area compatible with the broad area of hyperkeratosis with acanthosis [7]. ​Multiple brown pigmented dots arranged in a radial pattern at the periphery, resembling sunrays, along with regularly spaced brown dots and erythema in the surrounding non-lesional skin, have also been reported [8]. ​

Some other entities that present as flat-topped acral papules include verruca plana, flat warts, lichen planus, epidermodysplasia verruciformis, seborrheic keratosis, and Darier's disease (Table 1) [7]. ​Regarding the differential diagnosis with Darier's disease, AKV can be differentiated because it lacks the predilection for follicular areas and does not involve the oral mucosa and sebaceous regions [9]. ​The acral lesions in Darier's disease, though initially non-dyskeratotic, have every likelihood of developing dyskeratosis at a later age, whereas AKV remains non-dyskeratotic and non-acantholytic throughout life. Squamous cell carcinoma transformation may occur, although it is a rare phenomenon [10].​ Given its inheritance pattern, this condition follows a persistent, chronic course without remission [1]. 

**Table 1:** Differential diagnosis of acrokeratosis verruciformis of Hopf comparing morphology and topography of lesions as well as dermoscopic and histopathological findings The table was created by the authors using information from the cited sources [11-15]

	Acrokeratosis verruciformis of Hopf	Darier's disease	Epidermodysplasia verruciformis	Warts
Morphology	multiple flat-topped, skin-colored polygonal papules and verrucous plaques	brown, keratotic papules of sizes varying from pin-head to millet seed	The benign form is characterized by flat, wart-like, hypopigmented or hyperpigmented papules resembling tinea versicolor, whereas the malignant form presents as verrucous and seborrheic keratosis-like lesions	Small (1 to 4 mm), rounded or polygonal, skin-colored neoformations
Topography	Dorsal surfaces of the hands and feet	Seborrheic areas such as the forehead, central chest, back, and scalp margins	Trunk, sun-exposed surfaces, including the face, hands, and feet	Face and back of the hands
Dermoscopy	White network, cobblestone appearance, central white homogenous area	Central yellowish or brownish area with a whitish halo, Pinkish homogeneous structureless background, whitish scales surrounding the yellowish or brownish area	Fingerprint structures, hairpin vessels, or networklike structures	Papillomatous growth, bleeding spots, dotted and linear vessels, structureless yellowish-gray appearance
Histopathologic study	"Church spire" papillomatosis, acanthosis, hyperkeratosis, and hypergranulosis	Parakeratosis, hyperkeratosis, acantolysis; acantholytic cells and abnormal keratinocytes comprising corps ronds and grains	Mild acanthosis and hyperkeratosis	Hyperkeratosis with areas of parakeratosis, acanthosis, hypergranulosis, papillomatosis; koilocytes

The only effective treatment is superficial ablation [1].​ Other treatment strategies include cryotherapy, laser therapy, keratolytic solutions, and surgical excision. However, these are often associated with frequent recurrences [1]. ​Conservatively, keratolytic solutions and acitretin have been demonstrated to reduce the risk of malignant transformations [10]. 

## Conclusions

Our case report demonstrates the importance of suspecting a genodermatosis such as AKV in patients with a history of papular lesions and long-standing onychopathy. Darier's disease should be considered as the primary differential diagnosis. The diagnosis of AKV requires biopsy and histopathological study for confirmation. Appropriate management strategies should be initiated upon diagnosis to mitigate symptoms and prevent potential complications. This case is reported due to the unusual clinical manifestations and the absence of a family history of the disease. 
